# Nursing students’ perceptions of spiritual needs at the end of life. A qualitative study

**DOI:** 10.3389/fpsyt.2023.1132581

**Published:** 2023-07-14

**Authors:** E. Begoña García-Navarro, Sonia García Navarro, Luis Sousa, Helena José, María José Caceres-Titos, Ángela Ortega-Galán

**Affiliations:** ^1^Social Studies and Social Intervention Research Center & COIDESO, University of Huelva, Huelva, Spain; ^2^Department of Nursing, University of Huelva, Huelva, Spain; ^3^Escola Superior de Saúde Atlântica, Barcarena, Portugal

**Keywords:** spirituality, coping, end of life, nursing, student nursing, palliative care, mental health, wellbeing

## Abstract

Spirituality is defined as the meaning of life, being the very essence of life made up of all of the aspects inherent to it. During end-of-life processes, this need is shown to be particularly altered in patients and yet it is an aspect that the health professionals accompanying patients in this situation report being least equipped to address, alongside therapies that could help to meet these needs, such as art therapy. An exploratory qualitative study was conducted, adheres to the guidelines of COREQ (41). The study population were final year students undertaking a nursing degree at the University of Huelva, Spain. The sample was selected via intentional sampling using snowball recruitment from the study population. Stratification according to gender was performed due to the feminised nature of the population. Sample size was determined progressively during the research, with recruitment ceasing at 13 informants once information saturation was achieved. Inclusion criteria required that participants were to be final year students enrolled on a nursing degree who had provided consent to participate voluntarily in the research. The analysis Realized was interpretive phenomenological (IPA) as described by Smith (43–45). The present study revealed that students perceive their training on spiritual care to be deficient. Despite them reporting that they possess the skills and tools to provide end-of-life care, this is not enough to provide effective accompaniment, given that this moment brings them into touch with their own insecurities. Students verbalized the need to learn strategies to address this shortcoming regarding final accompaniment, for instance, through art, with creativity being one of the skills with the potential to uncover the meaning of life.

## Highlights

1. Nursing students place a lot of importance on accompaniment at the end of life, perceiving it to be a factor that attenuates fear and other prevailing emotions during this process contributing, in this way, towards mental wellbeing in the patient.
2. Students describe the importance of working in line with the patients’ spiritual needs but report not having adequate training to carry this out.
3. A patient’s spiritual needs at the end of life are related with coping during this process and students do not feel equipped to address this.
4. Academic training in the degree of nursing is currently not sufficient to meet the spiritual needs of patients during end-of-life processes and address their mental wellbeing at this stage through art therapy.

## 1. Introduction

Spirituality has been understood as the search for personal meaning that allows us to deepen our experiences, making our lives meaningful and, therefore, contributing to our happiness. It allows us to understand the world and the essence of things, helping us to understand the meaning of life and the human condition (1). Having defined this concept, it is important to highlight its complexity, emphasising that spirituality is an inherent component of human beings through which people seek meaning, purpose and transcendence, whilst also experiencing relationships with themselves, family, others, the community, society, nature, and that which is meaningful or sacred. Spirituality is expressed through beliefs, values, traditions, rituals, and practices (2). Appraisals concerning spiritual health, spiritual wellbeing and its existential and religious dimensions, allow us to consider the way in which the basic nature of human beings makes us both unique and transcendent. This gives all human beings the capacity to interact with their environment through two-way interpersonal relationships in which strengths and weaknesses are identified, opportunities are provided to grow and strengthen each other, and one is able to recognise, through their spiritual health, the support needed to face end of life processes (3, 4).

The spiritual dimension of human beings has become hugely important in health care in recent years, with more and more studies trying to study this phenomenon (5, 6). The holistic view of care favours the spiritual exploration of the sick person, thus enabling nursing interventions to be made available to address these needs. Spiritual care models are founded on valuing the spiritual dimension and training professionals in palliative and spiritual care so that they are able to listen to patients’ fears, dreams and pains and, subsequently, provide terminally ill patients with hope and validation (4, 7).

In order for professionals to care for patient needs regarding spirituality at the end of life, it is essential to deal with death and suffering by seeking an approach that meets the needs of the person and attends to transcendent aspects. Most importantly, an attitude of compassion towards the patient and their family is required, in addition to humility and openness towards understanding aspects of the process that are still little understood. This will enable patients to improve their capacity to help, grow in maturity and promote equity in the environment of suffering (8).

**Figure 1:**
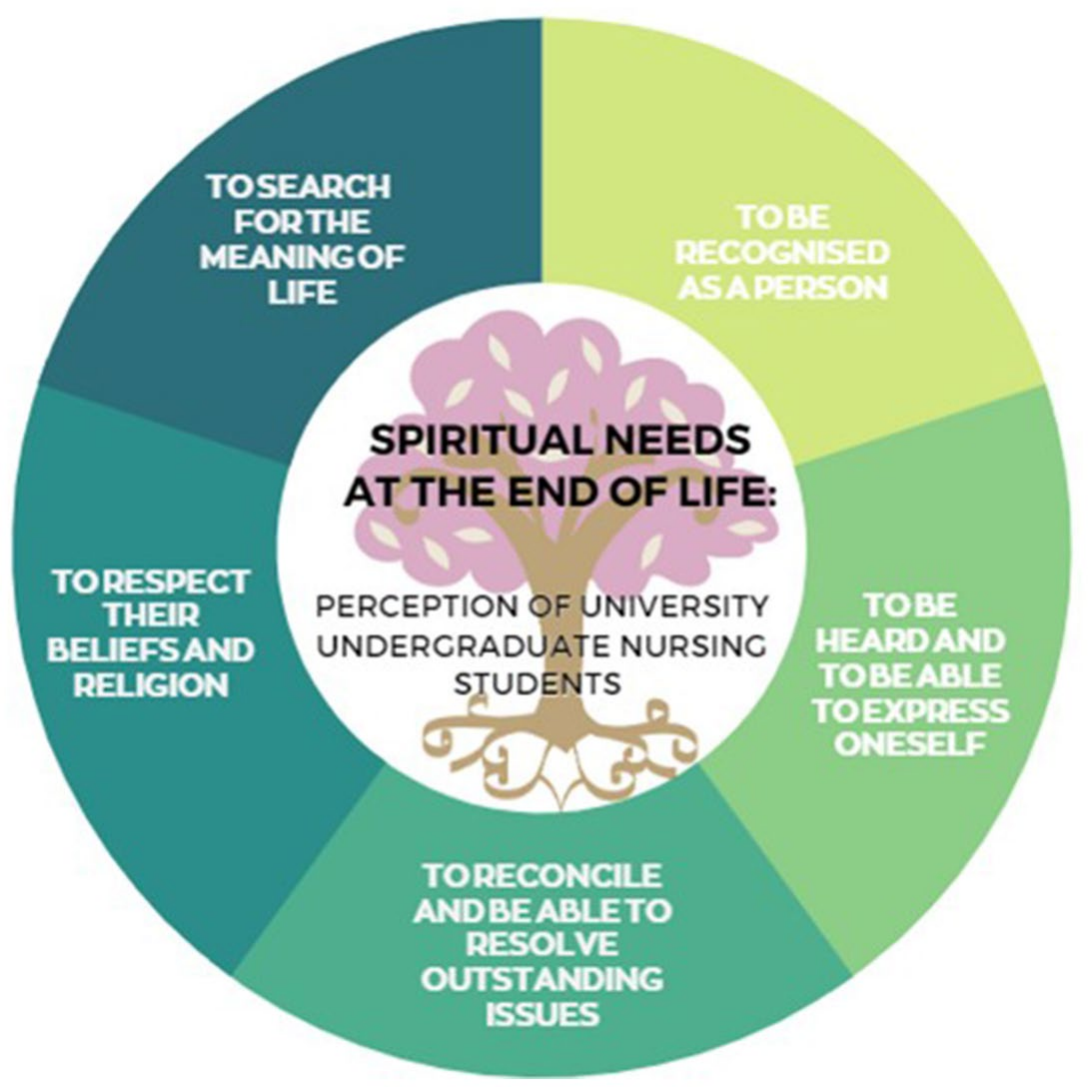
Spiritual needs at the end of life: perception of university undergraduate nursing students. Developed by the authors.

The spiritual care model has its foundations in the biopsychosocial-spiritual model (9) and the patient-centred care model (10), serving as a basis for explaining the main spiritual needs in the end-of-life process (11, 12). As shown in Figure 1, the approach to spiritual needs refers to the need to be recognised as a person, since illness threatens the integrity of the person in different areas: the individual self, the self as part of the family, the self as part of society, etc., and is often accompanied by a biographical rupture. Another need addressed in relation to this dimension is the need to find meaning in one’s existence and becoming, in other words, the search for meaning in life and suffering (13).

The need to free oneself from guilt and forgive oneself is another of the needs that should be addressed. Patients often analyse the terminal situation which generates internal conflict and frustration, fostering feelings of guilt. Another need that can be addressed is that of reconciliation, in other words, to feel forgiven. This need is more intense in terminally ill people, as there is a need to reconcile and resolve unresolved issues in order to close the circle of their existence and overcome resentment (14). On the other hand, the need to establish one’s life beyond oneself can manifest itself in two ways. The first is openness to transcendence, both ethical and religious, and the second is the need to rediscover a sense of solidarity (10); the need for continuity, for an afterlife. This need can be satisfied through offspring, work, enterprise or any type of achievement that can be relived. Another need that can be addressed is the need for authentic hope (11) and the need to express religious feelings and experiences. This need appears in all cultures and is often independent of the person’s religious orientation. Finally, the need to love and be loved is one of the most pressing, as it includes all the other needs mentioned above. This need is of vital importance as clinical experience reveals that, a person who loves and feels loved until the end, can die in peace (6).

The sick person has multiple needs—physical, emotional, social and spiritual—that can be projected through their own creativity and show them the meaning of their life. One way to address this individual dimension is through art therapy as it allows for a creative biographical approach that is particularly valuable at the end-of-life stage.

We have described the need to understand the transcendence of addressing these needs during the accompaniment of the person in the final phase of life or in moments of great vulnerability. The holistic vision of care integrates this approach as the essence of care (15). It is, therefore, necessary for both nursing professionals and nursing students to attend to spiritual needs and contemplate the human being in all of their complexity (16). However, despite the existence of different theoretical nursing models, such as Jean Watson’s Theory of Human Caring or Betty Neuman’s Systems Model that delve into this phenomenon of balance between body, soul and mind, both groups report a lack of knowledge on how to provide spiritual care (17).

Several authors (18, 19) conclude that end-of-life patients demand the spiritual component of care from professionals but are met with an ineffective response. To this end, it is necessary to improve the university training of nursing students and health professionals who are tasked with accompaniment as part of the final process (20). A study developed with nursing students from Jiangsu University, Zhenjiang, China (21) concluded that we should pay special attention should be paid to psychosocial and spiritual care teaching and preparing students to psychologically deal with the challenges in the process of patient’s dying. Coinciding with (22, 23) that affirm the need to address belief and spiritual needs in the academic curriculum of the degree in nursing, as well as ensuring that palliative care nursing is recognised and certified as a specialty in all European countries.

The health benefits of spirituality (24, 25) enable individuals to make beneficial lifestyle changes and become aware of the way in which beliefs, attitudes and behaviours can positively or negatively affect their health. This concept of spiritual well-being can be achieved through creative expressions such as art therapy (26), family rituals, meaningful work and religious practices. In this way, spirituality is configured as a personal tool that allows us to tackle the circumstances that we are faced with throughout life. This makes it a bedrock of intervention strategies delivered by health professionals.

Specifically, nursing requires not only technical skills but, also, skills related to all dimensions pertaining to the being of the person being cared for. It is, therefore, essential to consider the spiritual dimension, especially, in the final stage of life, where unpleasant feelings related to fear and anguish tend to develop (25), making it one of the most altered dimensions in this process (27). In this sense, various studies have shown that nursing plays an important role in coping with death (28–30), understanding the practice of spirituality as an instrument for the transformation and regulation of emotions (31).

Historically, nursing faculties have not been sufficiently aware of the need to address such needs, with the spiritual dimension only being mentioned exclusively in relation to specific subjects, such as palliative care. As interest in issues such as compassion, the helping relationship and spirituality has increased, universities are embracing this new health demand. In consideration of existing research (32–35), the University of Huelva is responding to the need to upskill future nursing professionals by addressing spiritual needs, not only in a conceptual way but, also, by incorporating a spirituality module within the subject of coping with death. The university is, in this way, a pioneer in achieving a holistic approach to care.

In consideration of other studies that have addressed this phenomenon (36–39), the aim of the present study was to uncover nursing student perceptions around spirituality and the way in which it is addressed through their clinical practice. The study will also identify possible areas for improvement, considering a phenomenon that has not yet been addressed, namely, self-knowledge (i.e., how nursing students’ own spirituality and search for personal meaning influences the care they provide) as a training need that is intrinsically linked to this concept.

## 2. Objectives

### 2.1. General objectives

Explore nursing student perceptions of the competencies needed to address spiritual care at the end of life.

### 2.2. Specific objectives

- Identify whether training received during nursing degree students is enough to deliver adequate spiritual care during end-of-life processes.
- Identify whether students perceive the need to allude to art as a strategy for addressing patient spiritual needs at the end of life.
- Describe the knowledge, practices and attitudes of nursing degree students with regards to spirituality.

## 3. Methodology

The objectives proposed by the present study enable the exploration, on the one hand, of the skills of nursing degree students regarding the spiritual component in patients going through the end-of-life process and, on the other hand, of the way in which students experience their own spirituality and how this enables them to accompany patients at this stage of their illness.

An exploratory qualitative study was conducted with a phenomenological approach. An in-depth examination was conducted of the experiences lived by students during accompaniment in end-of-life processes and the meanings attributed to this with respect to spiritual needs. Semi-structured interviews were conducted which enabled greater detail to be gathered from the descriptions given by participants of their experiences (40). The present research adheres to guidelines laid out by COREQ (41).

### 3.1. Study population

The selected sample was made up of nursing students who had studied palliative care or coping with death. This ensured that all participants had the same baseline regarding training on the issue under study. Initially, a convenience sampling approach was planned to target fourth year (final year) students who had received training on the end-of-life. Thus, the study was based on the validity and reliability of information provided by the selected population (36) and on the evidence produced by other similar studies (25, 42). In order to be considered for inclusion in the present study, students were required to meet criteria around training, be of adult age and voluntarily sign an informed consent sheet. The present study adhered to international ethical recommendations set out in the Declaration of Helsinki. All personal information provided was stored in compliance with legal requirements for the protection of personal data and the guarantee of digital rights (Organic Law 15/1999 of December 13th 1999 and Organic Law 3/2018 of December 5th 2018).

### 3.2. Data collection

Informants were recruited through collaboration with a class delegate who performed a leadership role for the project. This role served as a link to connect with potential candidates from which participants were selected and voluntarily agreed to conduct an in-depth interview. Participants were contacted by telephone by a project researcher who was experienced in this technique. After verifying that potential participants met pre-established criteria, they were summoned for interview, which was conducted by a different researcher. Interviews were conducted in a classroom in the faculty of nursing, considered a neutral space.

The entire procedure described above was recorded in field notes by a member of the research team. Interviews lasted a maximum of 70 min and always began with the same main question: do you feel prepared to accompany patients and meet their spiritual needs in the end-of-life process? Responses were not directed with informant freedom of speech being guaranteed. Where necessary, researchers re-centred the interview in line with a previously established interview guide until no new content emerged.

In order to ensure validity and reliability, the entire process of coding and discourse analysis was carried out independently by three members of the research team. One such member was external to the research and participated as a control for contact bias by participating only in data analysis. Discrepancies were discussed until a consensus was reached.

This triple-blinded approach aimed to minimise the implicit bias of physical contact between actors. A total of 13 in-depth interviews were conducted with 12 female students and 1 male student. This reflects the actual gender ratio in the student nurse population. Fieldwork was conducted between October and February 2021.

### 3.3. Data analysis

The analysis Realized was interpretive phenomenological (IPA) (43–45). The IPA emphasizes the study of personal experiences, focuses on facts that acquire great relevance for those who live them, for this, it formulates questions that suggest an in-depth exploration of the meanings built on these experiences. The stages of the analysis process were performed according to the model described by Smith. First of all the transcripts of the interviews and their reading, the emerging categories that arise from the speeches, the grouping of topics or lines of argumentation, the elaboration of the thematic tables and the writing of results.

Interviews were recorded and transcribed by the research team. These were reviewed on several occasions in order to make a first overall interpretation and to obtain general insights. Next, more in-depth analysis of the transcripts enabled discourse to be associated with student expressions of their lived and felt experiences of spirituality via the identification of codes and dimensions pertaining to common ideas as well as emerging categories.

Repeated use of the same codes-dimensions by different members of the research team (blind analysis) indicated that analysis got to the essence and exposed the meaning of the studied phenomenon.

Methodological rigour was achieved by following requisites for verification, validation and validity described by Meadows and Morse (46). Verification was achieved during the planning and informant recruitment phase, which also included the delegation of tasks to different members of the research team as described above. Validation was achieved through the different methods of data collection (interviews and observation of field notes). Data analysis and coding was done by the most experienced researcher and cross-checked by another team member. Internal validity of the study was achieved by cross-referencing with a research team member who did not belong to the same research group but had expertise in the subject matter.

## 4. Results

The final study sample at discourse saturation pertained to 13 fourth-year nursing students who had studied the subject of coping with death. Twelve were women and 1 was a man. The average age was 23.30 years. With regard to beliefs, 7 reported being atheists and 6 reported being Christian.

The basis of this research assumes that participants are an essential resource that allow reliable information to be obtained from which their perceptions and experiences in relation to the subject in question can be revealed. Initially, a number of main categories were proposed from the observation of field notes, with further categories emerging following examination of conducted interviews. Initial categories are shown in white, with additional categories emerging from interviews being shown in grey (Table 1).

**Table 1:** Description of analysed categories, codes and number of citations using ATLAS ti.

Dimension	Lines of argument	Codes	Citations
Spiritual care	Spirituality	Reason for being	8
		Meaning	7
		Knowledge and training	8
	Spiritual needs	Expressing their death	6
		Be at peace	6
		Respect	4
		Farewell	3
	Demand for spiritual care	Fear and insecurity	8
		Conversations with patients	7
	Care provision	Abilities	8
		Support	5
		Accompaniment	8
		Art therapy	5
End-of-life and their relationship with spirituality	Sickness and end-of-life	Positive influence	8
		Coping	8
		Greater transcendence	8
Ethical aspects	Religion	Relatedness	8
		Individuals without religious beliefs	8
	Beliefs and family traditions	Respect	7
		Quality care	6
		Coping	4
Aspects that reinforce spirituality	Resilience and reconciliation	Protection	8
		Coping	6
		Wellbeing	5
Obstacles to spirituality	Conspiracy of silence	Spiritual needs	8
		Fear to act	7
		Care challenges	6

Developed by the authors.

Following analysis of informant data, eight lines of argument are established as the main bedrock of the discourse. Each of these lines of argument is described using the codes or categories that define them, in addition to quotations extracted from analysis. Shaded sections pertain to categories that emerged from informant discourse.

The first line of argument discusses the need to understand the concept of spirituality perceived by those interviewed. This construct is described as an individual’s reason for being (a category emanating from actual discourse) and their search for meaning. It is related with the individual’s social and moral network, with having a sense of hope and purpose in life, enabling them to forgive and be forgiven. This coincides with the theoretical construct laid out in Figure 1.

E5: “*I believe that spirituality is the essence of each person, their reason for being.*”

E10: “*For me spirituality is what you are, the meaning that people give to things and how you live your life.*”

As professionals, in order to be able to deliver care in this context it is necessary to count on key knowledge. Such knowledge was considered by those interviewed to be in short supply during university training, with students stating that, although this skill should be developed transversally, it was only tackled as part of an optional module (“coping with death”) during the fourth year. As it was not a compulsory module, not everybody opted to take it, meaning that many students finished their studies without any training on this topic.

E7: “*I have no knowledge whatsoever of how I have to care for or deal with a person’s spirituality. It has only been covered in one part of one subject. We should not finish our studies without knowing how to deal with this*.”

E11: “*Sometimes I have been next to a patient who was leaving us and I have not known how to tell them how I felt, how to express to them that I was there to accompany them, I lack this know-how* … *we need more specific training to know what the person needs at any given moment.*”

Given that presented, although participants knew how to define the concept, the lack of knowledge made it difficult for them to identify **patients’ spiritual needs**. This formed the second line of argument identified by the present study.

In the case of patients who find themselves in terminal situations, the need to be at peace with oneself and others was highlighted, in addition to the need to feel respected throughout the entire process until the moment at which life ends. This demands the ability to express the way in which one wishes to die, making it easier to say goodbye.

E4: “*Not all professionals have that compassionate outlook that is so necessary. It is necessary to understand what worries the individual: family, money, feeling a burden* … *so that they can leave in peace, with nothing left to say or do, favouring the process of saying goodbye.*”

The third line of argument, the **way in which patients make spiritual demands**, has a dual nature. On the one hand, those interviewed stated that these demands caused them fear and insecurity, due to them not having the tools and knowledge required to address this spirituality. On the other hand, patients reported feeling a sense of satisfaction at feeling needed by patients. In order to address this need, holistic and comprehensive care should be provided to patients, favouring active listening and the expression of feelings.

E9: “*I would feel insecure, because I think I am not able to deal with it. I am afraid of the unknown, but surely with the passage of time I would be more confident and so would the patient* … *then* … *their days would be better* … *I think that would be a win–win situation for both parties. Because afterwards that fear would become satisfaction at being able to help them.*”

E1: “*Talking to the patient alone, telling them that I am not there to judge them or mistreat them, or make them feel bad, but that I am there to help. If that person really has a conflict, talking to them can make them come out and be the one who communicates their concerns to us.*”

In order to address this need, holistic and integrated individual care must be provided. Strategies such as active listening and the expression of feelings should be encouraged.

The nursing interventions alluded to above make up the fourth line of argument, specifically, referring to the **spiritual care offered by nursing professionals**. Despite being an incredibly broad category, informants emphasised the importance of nursing professionals at the time of perceiving demands from sick individuals for spiritual care. In this sense, informants believed that nurses could offer spiritual care through a variety of abilities such as listening to patients and giving them time to discuss and explore their fears, anxieties and problems. For this, it is necessary to speak with the patients and earn their trust. This enables the patient to discuss their concerns with the nurse, feeling supported by it. Such trust can be earned by accompanying the patient and providing spiritual care. In other words, always taking into consideration what the patient thinks and feels. Types of care provided by nurses that were mentioned included active listening and providing support, encouragement and strength.

E7: “*Being there, offering them support, being empathetic* … *often simply listening to them is enough and we manage to make them feel calmer.*”

E12: “*Have in mind that the spirituality of the patient has quite an influence of accompaniment because, at the end, it is like another aspect in itself of the person, so if you are not considering spirituality, you are leaving a part of their needs* “*uncovered*” *and, at the end of the day, nursing tries to cover all needs, whether they are apparent or not.*”

When students were asked about patient care and whether they feel that they manage to instil spiritual harmony in patients through art, most reported the need to avoid unpleasant thoughts by providing patients with options. However, although students showed favourable attitudes towards meeting patient needs and reported a desire to possess the knowledge required to be able to offer quality spiritual care, they do not know how to use techniques to help patients clarify their beliefs and values. Such techniques could include the use of meditation, relaxation and guided imagery; however, students instead tend to rely on other resources drawn from their own experiences.

E3: “*When I am at university, far away from my home and family, and I feel homesick or melancholic, I start drawing. This makes me feel close to my family, so maybe I would try to give this resource to the person who needs or wants it.*”

It is clear that the satisfaction of patient spiritual needs positively influences coping with illness, emphasising the importance of nursing as a means to satisfying these needs. The **relationship that exists between illness and end-of-life** makes up the fifth line of argument. Over the course of the interviews, it was mentioned that patients’ spiritual needs are there throughout their lifetime, however, at the end of life spirituality and these needs take on greater importance, whilst also being different to the needs of an individual who does not know that the end is upon them. In this sense, becoming aware of one’s own death allows one to reflect on present needs, which, with the collaboration of health professionals, will try to be covered to achieve the end of life desired by the person.

E3: “*I think that one’s spiritual state has quite an influence, I would change their attitude, their way of seeing things, of communicating with their family, the way of seeing life in a different way and it helps you to try to adapt and accept what you have. If these spiritual needs are covered then one can cope better with illness.*”

This greater intensity in end-of-life situations leads patients to recap all of the stages of their life, which can lead them to experience a lot of pain. In order to manage this, the sixth line of argument refers to **religion** and its relationship with spirituality. Over the course of the interviews, it was reflected that students were aware of an existing relationship between both of these aspects, mentioning that religion is included as one of the spiritual needs that can be present in patients. These students commented that nurses should be able to provide spiritual care by arranging meetings with the hospital chaplain, priest, pastor, rabbi or spiritual leader, where relevant to the patient.

E1: “*For many people, spirituality and religion go hand in hand, thinking that when they die they will go to a better world, that they will be with relatives, that God will protect them*….” *I think that can help a lot of people to cope with problems and* … *whatever comes their way in life.*”

A distinction is therefore made between the two terms, transcending spiritual care beyond religion and also being applicable to atheists and agnostics.

E11: “*Spirituality is a very broad concept, you can be spiritual and an atheist. It is achieving internal harmony through the things that make you happy and one of those things may or may not be religion.*”

Besides religion, other important aspects in the lives of individuals exist that are related with spirituality, such as **beliefs and family traditions**. These aspects make up the seventh line of argument. Our family heritage at birth configures a set of values, life principles and norms, beliefs and traditions, as a part of our ancestral setup. Family is a unique space characterised by acceptance and love for those who enter it, giving members a sense of belonging. Each family member plays an important role as life plays out. Informants agreed that keeping in mind and respecting these beliefs and family traditions would enable better accompaniment of the ill patient, making it possible for them to deliver quality nursing care. The majority believe that nurses are capable of delivering spiritual care whilst respecting patient privacy, dignity and religious and cultural beliefs.

It was mentioned that all of these aspects lead to better coping with illness, which will lead to the satisfaction of spiritual needs within the individual.

E4: “*I believe that they have a big influence because it is what has formed you as a person and sharing that with your family means that they person lives better* … *because if you do not respect these traditions or beliefs, then the person will not be well cared for or attended* … *so then it would be useful if we knew what these traditions or beliefs are to be able to respect them and for the patient to feel better* … *because if we do not do that then spirituality will end up being compromised.*”

The eighth line of argument refers to **resilience and reconciliation** and the way in which these affect spirituality. The former term is taking on increasing importance with regards to the maintenance of good health and psychological wellbeing in an ever more dynamic and challenging environment. Resilience shields mental health during times of sickness, enabling sickness to be overcome and leading to better spiritual wellbeing in the patient.

E1: “*Resilience is* … *constant and personal growth, knowing how to recognise when something that affects you can happen again and you learn from the situations you are given in life. I think it’s very important because it makes you learn from problems and be able to help deal with them and solve them in a better way* … *or in the most positive way you can.*”

With regards to reconciliation with one’s own issues, in other words, forgiving and being forgiven, this is one of the most important needs to arise in end-of-life patients. Due to the proximity of death, the need to reconcile with themselves, resolve pending issues, bring an end to the circle of their existence and overcome resentment becomes more urgent. Over the course of the interviews, aspects were mentioned such as saying goodbye, which is perceived as an indispensable part of reconciliation. Further, family is held in high esteem, given that reconciliation allows relatives to be more at peace, whilst the individual themself can also be more at peace with themselves, achieving wellbeing.

*E5*: “*Reconciliation is going to help them leave more at ease, thinking that they have solved the problem they were worried about. It allows you to leave in peace, satisfied.*”

Nonetheless, the expression of feelings and resolution of outstanding conflicts is not always favoured, as patients sometimes fall into a **conspiracy of silence**. This forms the ninth and final line of argument. Informants were all in agreement that the conspiracy of silence negatively impacts the accompaniment provided by professionals. It causes professionals to tread carefully out of fear when dealing with patients and impedes them from delivering quality care. In addition, this argument reasons that the conspiracy of silence prevents spiritual needs from taking on greater importance and, likewise, stops these needs from being met.

E9: “*If you want to help someone to die well, peacefully, and that person is not aware of what they have and what is happening to them, that’s a pretty big bump in the road. If you are not taking spirituality into account, you are leaving a part of their needs* “*unmet.*”

This causes professionals to act with fear of approaching the patient, not knowing what to say or do, and prevents quality care from being carried out.

E8: “*Professionals already tread with the fear of* … *thinking about what they have said, what they have not* … *because the patient is going to ask you questions and you have to take a bit of a step back. The professional is not going to know how to manage this and in reality, what it does is affect the patients themselves more* … *and it is going to mean that we cannot provide them with sufficient care or the care that they really need.*”

Students also refer to the fact that it prevents spiritual needs from taking on real importance and, therefore, prevents them from being met.

E4: “The patient, not having all the information, does not know what is happening to them, does not know if they will survive or not…. This prevents the development of that spirituality, makes it difficult for their needs to be met. Because your needs, knowing that you are going to die, may be different. The patient must have complete information so that they themselves can know how they feel, what they need and what they want.”

## 5. Discussion

End-of-life accompaniment must include care that is focused on patient spiritual needs, in such a way that the patient also perceives their needs to be met in the process. Various authors (14–16, 29–31) agree that tackling the end-of-life by considering spiritual needs through care or different strategies, such as art therapy, lead to better acceptance of the severity of illness. Patients who feel accompanied on a spiritual level recover their dignity and this, at the same time, influences family wellbeing (18, 19). This idea coincides with perceptions reported by the study sample, from which it was observed that those who verbalise the importance of meeting this need, did not consider equipping themselves with specific training until the time of end-of-life, nor did they consider informing themselves about the different therapies used to tackle this process, such as art therapy (47).

With regards to the main aim of the present research which was to identify student perceptions of spirituality and its care, students described the difference between the concepts of spirituality and religion. They referred to the more clinical acceptance of these terms and highlighted that it is possible to provide spiritual care to individuals regardless of their faith. In accordance with that reported by authors such Fernández et al. (48), the present study identified that it is common practice to combine the two concepts of spirituality and religion as a single reference to the same patient need, particularly, in the final moments of life when patients cling to their beliefs, whether they are religious or not. A study carried out in universities in Portugal and Brazil (49) again agree on the need to address these differences with students; both groups of students (Portugueses and Braziliam) indicated they should be prepared to address religiosity and spirituality with patients, that these subjects should be included in the curriculum and that they were not properly prepared to address spiritual issues.

Students identified the need to respect the beliefs and decisions of individuals, together with the need to be able to provide solutions to clarify any confusion that patients may have in this process in order to provide them with an end that is adapted to their needs.

Informants described the skills required to address spirituality at the end of life. In accordance with various authors (50–54), they highlighted the importance of working on compassion, listening, empathy, respect and worry. This will enable a climate of trust to be established between the nurse and the patient, leading to a greater degree of satisfaction in patients and families. The study sample referred to not addressing this need due to the fear it caused them and not having the necessary tools. In addition, they referred to the need for more training on these abilities, alongside diverse strategies to work with these patients during this phase, such as art therapy (47).

Given the need for more training was reflected in the discourse of all participants, leading to information saturation by the fourth interview, a dimension was included that pertained to having enrolled on the optional module, Coping with Death, as part of the nursing degree at the University of Huelva. This generated responses that touched on the capacity to deliver accompaniment characterised by compassion and listening, in addition to the development of greater creativity (art therapy) at the time of conducting interviews with patients who found themselves at the end of life and their families. Reference was also made to projects carried out during the development of the subject as a facilitating factor in the acquisition of these competences. This coincides with the research developed by Cano and Sáenz Castro (54) in which the realization of research projects in the classroom were an alternative that allowed the development of higher thinking skills, as well as learning for life and values to accompany in the final process.

Students were aware of the importance of spirituality, although discourse evidenced a lack of training on the topic and a failure to tackle the issue through the academic nursing degree course (in the case of those who had not enrolled on specific training). Participating students emphasised the need to modify what was on offer academically through modules focused on end-of-life care. This is supported by findings reported by Bennet and Thompson (55), from a study conducted with students from the University of Liverpool in the United Kingdom.

Present findings describe the way in which students who have received specific end-of-life training recognise the importance of accompanying patients during the final process, helping them to resolve pending issues and overseeing mediation with relatives to ensure dignity during this stage given that it forms part of their final wishes in relation to their spiritual needs. This argument emerged over the course of interviews and has also been highlighted by authors such as (33, 34, 36, 37). Students reported a need for professionals to be equipped with tools that allow patients to be able to reconcile themselves with their personal affairs, recognising the huge magnitude of this need when it comes to enabling a “good death,” in addition to enabling families to cope and grieve in a more positive way.

The present study coincides with previously conducted research (47, 56), with regards to identification of factors such as the conspiracy of silence (53). This factor acts to prevent health professionals and students from delivering quality end-of-life care, leading to deficient accompaniment. This will mean that the spirituality and spiritual needs of the individual receiving care will be compromised. The sick person themselves, their family and implicated professionals face serious challenges when it comes to speaking openly about spiritual needs given that these are associated with imminent death. Nonetheless, when the opportunities to communicate are available, both the sick person and their relatives can be at peace and find a serenity that is wholly comforting (57). For this reason, participating nursing students considered it important to be able to obtain the tools needed for professionals to be able to talk with the patient and their families. Such communication enables an agreement to be reached around the information that both sides would like to receive, emphasising the importance of this for coping with the process and, in this way, benefitting the spiritual state of the patient.

The present sample emphasised the value of accompanying patients in this process. In fact, some of the discourse recorded identified this as an attenuating factor when it comes to fear of the unknown. This finding coincides with those reported in other studies (30, 31). These authors identified spiritual care models as providing a reference framework through which health professionals can connect with their patients, listen to their fears, dreams and pains, collaborate with their patients as allies in their care and provide, via the therapeutic relationship, the chance for healing. Canteros (58) in a recent study by the University of Chile concludes as spiritual accompaniment provides comprehensive and holistic healthcare, which generates multiple clinical benefits, humanizing and dignifying health care. Thus, accompaniment creates the required climate of trust between the professional and patient for enabling the type of physical and psychological comfort which allows the end of life to occur with dignity.

## 6. Limitaciones

To minimize investigator bias, COREQ (41) criteria were used.

The use of the snowball selection method could have introduced bias, however, new topics—emerging categories—were identified from the data, adding meaning to the data confirmed by the conceptual framework, this gives us security of the heterogeneity of the discourse analyzed.

The students subject to study are trained in a subject where spiritual needs are addressed, that may imply the competences described in some of the results, probably not coinciding in other populations where this specific training is lacking, although this characteristic has been taken into account throughout the content analysis and in the description of the findings.

## 7. Conclusion

The present study enabled identification of the deficient training received by nursing students on spirituality, despite the fact that they perceive themselves to have the necessary skills and tools to address it (empathy, presence, active listening, compassion). At present, nurses report acquiring their skillset from the training received from the academic course. Students verbalise the need to learn strategies for approaching end-of-life accompaniment through art therapy. Creativity is a manifestation of the inner harmony of human beings that allows connections to be formed with oneself and others. This ability allows individuals to get in touch with the meaning of life.

However, for some professionals who accompany people in their end-of-life process, a relationship is established that favours addressing spiritual needs despite the lack of specific training on spirituality. This is due to the therapeutic relationship established with the vulnerable patient and the innate need of the professional to accompany in a compassionate way.

The particularity of this accompaniment allows some professionals to develop innate skills such as presence, respect for silence (favouring introspection of the accompanied person), active listening, compassion and, even, the promotion of moments of relaxation despite not having been trained to do so. All of these skills make it possible to continue accompanying the vulnerable person in a better way during times of suffering. At the same time, it provides professionals with strategies to identify their own spiritual needs and become aware of their existential inner world in order to provide spiritual care.

The present study provides evidence of the need for training in these aspects, not only with regard to spirituality but, also, with regards to end-of-life care.

In relation to the implications of the study, it is important to consider a greater focus on how practice and school can collaborate for the best learning outcome and how nurse managers can play an active role in this.

The priorities identified here should be used to guide future spiritual care research and clinical and educational initiatives.

## Data availability statement

The original contributions presented in the study are included in the article/Supplementary material, further inquiries can be directed to the corresponding author.

## Ethics statement

The studies involving human participants were reviewed and approved by Ethics Committee PPEIBA, government of Andalusia, Spain (protocol code 01/2020 CEPP and date of approval 18/01/2020). The patients/participants provided their written informed consent to participate in this study. Written informed consent was obtained from the individual(s) for the publication of any potentially identifiable images or data included in this article.

## Author contributions

EG-N was responsible for coordination, study design, analysis, data interpretation, and writing of the manuscript. SN participated in fieldwork, study design, analysis, data interpretation, and writing of the manuscript. EG-N, SN, LS, HJ, MC-T, and ÁO-G, participated in data interpretation and writing of the manuscript. All of the Spanish researchers participated in the translation of the interview guide and interview delivery. The international team (LS and HJ) conducted the double-blind analysis. All authors contributed to the article and approved the submitted version.

## Conflict of interest

The authors declare that the research was conducted in the absence of any commercial or financial relationships that could be construed as a potential conflict of interest.

## Publisher’s note

All claims expressed in this article are solely those of the authors and do not necessarily represent those of their affiliated organizations, or those of the publisher, the editors and the reviewers. Any product that may be evaluated in this article, or claim that may be made by its manufacturer, is not guaranteed or endorsed by the publisher.
