# Supplementary material for: Nursing students’ perceptions of spiritual needs at the end of life. A qualitative study
Source: Front Psychiatry. 2023 Jul 14;14:1132581. doi: 10.3389/fpsyt.2023.1132581 (PMC10375720; doi:10.3389/fpsyt.2023.1132581)
Supplement: Supplementary file 1 [file Table_1.docx]

**SUPPLEMENTARY MATERIAL**

**Table 1. Participant sociodemographic characteristics.**

| Participants | Age | Sex | Beliefs | Knowledge on end-of-life |
| --- | --- | --- | --- | --- |
| E1 | 25 | F | Christian | Yes |
| E2 | 27 | F | Christian | Yes |
| E3 | 22 | F | Christian | Yes |
| E4 | 26 | F | Atheist | No |
| E5 | 22 | F | Atheist | No |
| E6 | 22 | F | Atheist | No |
| E7 | 29 | F | Christian | Yes |
| E8 | 21 | F | Atheist | No |
| E9 | 23 | F | Christian | No |
| E10 | 21 | F | Atheist | Yes |
| E11 | 21 | F | Atheist | Yes |
| E12 | 23 | F | Christian | No |
| E13 | 21 | M | Atheist | No |

Source: Developed by the authors.

**Table 2. Description of analysed categories, codes and number of citations using Atlas ti.**

| Dimension | Lines of argument | Codes | Citations |
| --- | --- | --- | --- |
| Spiritual care | Spirituality | Reason for being | 8 |
|  |  | Meaning | 7 |
|  |  | Knowledge and training | 8 |
|  | Spiritual needs | Expressing their death | 6 |
|  |  | Be at peace | 6 |
|  |  | Respect | 4 |
|  |  | Farewell | 3 |
|  | Demand for spiritual care | Fear and insecurity | 8 |
|  |  | Conversations with patients | 7 |
|  | Care provision | Abilities | 8 |
|  |  | Support | 5 |
|  |  | Accompaniment | 8 |
|  |  | Art therapy | 5 |
| End-of-life and their relationship with spirituality | Sickness and end-of-life | Positive influence | 8 |
|  |  | Coping | 8 |
|  |  | Greater transcendence | 8 |
| Ethical aspects | Religion | Relatedness | 8 |
|  |  | Individuals without religious beliefs | 8 |
|  | Beliefs and family traditions | Respect | 7 |
|  |  | Quality care | 6 |
|  |  | Coping | 4 |
| Aspects that reinforce spirituality | Resilience and reconciliation | Protection | 8 |
|  |  | Coping | 6 |
|  |  | Wellbeing | 5 |
| Obstacles to spirituality | Conspiracy of silence | Spiritual needs | 8 |
|  |  | Fear to act | 7 |
|  |  | Care challenges | 6 |

Source: Developed by the authors.

**Table 3. Codes and nursing student discourse about the concept of spirituality.**

| Line of argument | Codes | Citations | Discourse |
| --- | --- | --- | --- |
| Spirituality | Reason for being | 8 | E5: *“I believe that spirituality is the essence of each one, a person’s reason for being”.* |
|  | Meaning | 7 | E10: *“For me, spirituality is what you are, the meaning that people attribute to things and the way they live life”.* |
|  | Knowledge and training | 8 | E7: *“I don’t have any knowledge about how I have to care for or address a person’s spirituality. Only one part of the subject has dealt with the topic. We shouldn’t finish our studies without knowing how to address that”.* |

Source: Developed by the author

**Table 4. Codes and nursing student discourse on the spiritual needs perceived by students pertaining to patients in terminal situations.**

| Line of argument | Code | Citations | Discourse |
| --- | --- | --- | --- |
| Spiritual needs | Expressing death | 6 | E2: *“Everybody should write a few last wishes… how they would like to die… write a few last words, how they would like to say goodbye”.* |
|  | Being at peace | 6 | E11: *“I think that a person who is ok with themselves and their family will have a more peaceful and tranquil death”.* |
|  | Respect | 4 | E6: *“To feel respected, because that need is always there, whether you die or not, and in this aspect us nurses can do a lot”.* |
|  | Farewell | 3 | E10*: “I think that saying goodbye is a really important need, because bringing an end to stages and having the chance to say goodbye to your loved ones is really necessary as, in that way, you leave all of your issues settled and not a single complication is left, that way you can move on more easily”.* |

Source: Developed by the authors.

**Table 5. Codes and nursing student discourse about patient demands for spiritual care**

| Line of argument | Codes | Citations | Discourse |
| --- | --- | --- | --- |
| Demand for spiritual care | Fear and insecurity | 8 | E9: *“I would feel really insecure, because I don’t think I am able to tackle it. I have a fear of the unknown but, surely, with the passing of time I would be more confident and the patient would too… as a result… their days will be better… I think that, that way, they win both ways because, afterwards, this fear becomes satisfaction at being able to help them”* |
|  | Conversations with the patient | 7 | E1*: “To speak alone with the patient, to tell them that I am not here to judge them or mistreat them, nor to make them feel bad about themselves, that I am there to help. If this person really has a conflict, talking with them can get them to come out of their shell and take the lead in communicating their concerns”.* |

Source: Developed by the author

**Table 6. Codes and nursing student discourse about the care offered by professionals**

| Line of argument | Codes | Citations | Discourse |
| --- | --- | --- | --- |
| Care provision | Abilities | 8 | E7*: “Being there, offering them support, being empathetic… Often simply listening to them is enough and we manage to make them feel calmer”.* |
|  | Support | 5 | E3: *“Communicate with the patient, respect them at all times and give support both to them and to their relatives, like with any person you are accompanying”.* |
|  | Accompaniment | 8 | E12: *“Have in mind that the spirituality of the patient has quite an influence of accompaniment because, at the end, it is like another aspect in itself of the person, so if you are not considering spirituality, you are leaving a part of their needs “uncovered” and, at the end of the day, nursing tries to cover all needs, whether they are apparent or not”.* |
|  | Art therapy | 5 | E6: *“I would try to support them and accompany them to meet their needs, make them more open to the process… put the television or music on for them, or get them a book from the library”.* |

Source: Developed by the authors.

**Table 7. Codes and nursing student discourse about the concept of spirituality.**

| Line of argument | Codes | Citations | Discourse |
| --- | --- | --- | --- |
| Sickness and end-of-life | Positive influence | 8 | E11:*“Covering all spiritual needs can positively influence the illness, given that this has a large effect at a personal level and effects your mood state… That way, being good spirituality and having a clean conscience, being calm and viewing the process as something natural, I think, has a good influence”.* |
|  | Coping | 8 | E3: *“I think that one’s spiritual state has quite an influence, I would change their attitude, their way of seeing things, of communicating with their family, the way of seeing life in a different way and it helps you to try to adapt and accept what you have. If these spiritual needs are covered then one can cope better with illness”.* |
|  | Greater transcendence | 8 | *“Spirituality is something that you carry inside but it manifests at different times of your life to a different extent. At the end, we all go through it. Right? We will die and start to think about a load of things, like what there is after, what will happen to my body, what will happen to my close ones, to my soul…* |

Source: Developed by the authors.

**Table 8. Codes and nursing student discourse about the influence of religion on spirituality.**

| Line of argument | Codes | Citations | Discourse |
| --- | --- | --- | --- |
| Religion | Relatedness | 8 | E1: *“For many people, spirituality and religion go hand in hand, the thinking that when they die they will go on to a better world, they will be with relatives, that God will protect them…” I think that this can help many people cope with their problems and… everything that they have coming to them in life”.* |
|  | People without religious beliefs | 8 | E12: *“I think that you can have spirituality, regardless of your religion… or if you are atheist. In both cases, spirituality is there and spiritual needs are too, they just might be different”.* |

Source: Developed by the authors.

**Table 9. Codes and nursing student discourse about beliefs and family traditions.**

| Line of argument | Codes | Citations | Discourse |
| --- | --- | --- | --- |
| Beliefs and family traditions | Respect | 7 | E4*: “I believe that they have a big influence because it is what has formed you as a person and sharing that with your family means that they person lives better… because if you don’t respect these traditions or beliefs, then the person won’t be well cared for or attended… so then it would be useful if we knew what these traditions or beliefs are to be able to respect them and for the patient to feel better... because if we don’t do that then spirituality will end up being compromised”.* |
|  | Quality care | 6 | E7: “When we deliver care we must consider these traditions because rituals and customs have moments when they greatly reveal this spirituality*”.* |
|  | Coping | 4 | E9: *“I can’t abandon the person at this time nor make them feel bad, much less because almost certainly that person is clinging on to this “tiny thread of hope”. Because if the person sees that their beliefs and traditions are not being affected, they will feel better spiritually… this will help them deal better with sickness, as it is one thing that leads to the other”.* |

Source: Developed by the authors.

**Table 10. Codes and nursing student discourse about resilience and reconciliation.**

| Line of argument | Codes | Citations | Discourse |
| --- | --- | --- | --- |
| Resilience and reconciliation | Protection | 8 | E12: *“I think that to be good with oneself, if you are a resilient person, if you are a person who more or less knows how to deal well with issues, I think that resilience has a large effect or intervenes with the spirituality of a person”.* |
|  | Coping | 6 | E1: “*Resilience is… constant and personal growth, knowing how to recognise when something affects you that it can happen again and you learn from situations that happen in life. It seems very important to me because it makes you learn from problems and helps you cope with them and resolve them in a better way… or in the most positive way you can”.* |
|  | Wellbeing | 5 | E5: *“It will help them to go on more comfortably, thinking that they have settled the issue that was worrying them. It allows you to go in peace, satisfied”.* |

Source: Developed by the authors.

**Table 11. Codes and nursing student discourse about the conspiracy of silence.**

| Line of argument | Codes | Citations | Discourse |
| --- | --- | --- | --- |
| Conspiracy of silence | Spiritual needs | 8 | *E4: “The patient, as they do not have all of the information, doesn’t know what is happening to them, they don’t know if they will survive or not… This prevents them developing this spirituality, it makes it difficult for their needs to be satisfied. Because your needs knowing that you are going to die can be different. Patients must have all the information so that they themselves can know how to feel, what it is that they need and what it is that they want”.* |
|  | Fear to act | 7 | *E8: “Professionals are already going around with the fear of… let’s see what’s been told to them, what hasn’t… because the patient is going to ask you things and you have to hold back a little. The professional is not going to know how to conduct themselves and, really, what it does it affect more the patients themselves… and that is going to mean that we can’t provide enough care or the care that they really need”.* |
|  | Challenges with care | 6 | *E9: “If you want to help somebody have a good, calm death, and that person is not aware of what they have and what is happening to them, it is a pretty big speedbump that you find in your way. If you are not taking spirituality into account, you are leaving one part of their needs “uncovered”.* |

Source: Developed by the authors.
